# Enhanced Vulnerability of Diabetic Mice to Hypervirulent *Streptococcus agalactiae* ST-17 Infection

**DOI:** 10.3390/pathogens12040580

**Published:** 2023-04-11

**Authors:** Jéssica da Conceição Mendonça, João Matheus Sobral Pena, Noemi dos Santos Macêdo, Dayane de Souza Rodrigues, Dayane Alvarinho de Oliveira, Brady L. Spencer, Eduardo José Lopes-Torres, Lindsey R. Burcham, Kelly S. Doran, Prescilla Emy Nagao

**Affiliations:** 1 Laboratory of Molecular Biology and Physiology of Streptococci, Institute of Biology Roberto Alcantara Gomes, Rio de Janeiro State University, Rio de Janeiro 20550-013, RJ, Brazil; jess.cmendonca@gmail.com (J.d.C.M.);; 2 Department of Microbiology, University of Tennessee Knoxville, Knoxville, TN 37916, USA; lburcham@utk.edu; 3 Laboratório de Helmintologia Romero Lascasas Porto, Department of Immunology, Microbiology e Parasitology, Rio de Janeiro State University, Rio de Janeiro 20550-013, RJ, Brazil; 4 Department of Immunology and Microbiology, University of Colorado School of Medicine, Anschutz Medical Campus, Aurora, CO 12800, USA

**Keywords:** *Streptococcus agalactiae*, Group B *Streptococcus*, diabetes *mellitus*, bacterial dissemination

## Abstract

*Streptococcus agalactiae* (Group B *Streptococcus*, GBS) is the leading cause of neonatal sepsis and meningitis but has been recently isolated from non-pregnant adults with underlying medical conditions like diabetes. Despite diabetes being a key risk factor for invasive disease, the pathological consequences during GBS infection remain poorly characterized. Here, we demonstrate the pathogenicity of the GBS90356-ST17 and COH1-ST17 strains in streptozotocin-induced diabetic mice. We show that GBS can spread through the bloodstream and colonize several tissues, presenting a higher bacterial count in diabetic-infected mice when compared to non-diabetic-infected mice. Histological sections of the lungs showed inflammatory cell infiltration, collapsed septa, and red blood cell extravasation in the diabetic-infected group. A significant increase in collagen deposition and elastic fibers were also observed in the lungs. Moreover, the diabetic group presented red blood cells that adhered to the valve wall and disorganized cardiac muscle fibers. An increased expression of KC protein, IL-1β, genes encoding immune cell markers, and ROS (reactive oxygen species) production was observed in diabetic-infected mice, suggesting GBS promotes high levels of inflammation when compared to non-diabetic animals. Our data indicate that efforts to reverse the epidemic of diabetes could considerably reduce the incidence of invasive infection, morbidity and mortality due to GBS.

## 1. Introduction

*Streptococcus agalactiae*, also known as Group B *Streptococcus* (GBS), is a commensal bacterium colonizing the intestinal and genitourinary tracts in 15–30% of healthy adults; however, it remains one of the most important invasive pathogens in infants and the elderly [1]. The number of infections in non-pregnant adults with chronic medical conditions, particularly diabetes *mellitus*, has been increasing worldwide. Previous studies showed that diabetes was present in 40.1% of patients with invasive infection by GBS, including soft-tissue infections, necrotizing fasciitis, cellulitis and mediastinal, subcutaneous, and multiple muscular abscesses [2,3]. In addition, pregnancies complicated by gestational diabetes have been found to have higher rates of colonization with GBS than pregnancies without diabetes [4].

The prevalence of diabetes has risen markedly in the world [5]. Bacterial infections are a relatively frequent occurrence in diabetic patients and are associated with increased morbidity and mortality [6,7]. Altered immune function in patients with diabetes *mellitus* leads to an increased risk of multiple types of infections. People with diabetes are three times more susceptible to developing bacterial sepsis and suffer far worse outcomes [8]. They also have a higher risk of developing pneumonia, diabetic cardiomyopathy, blood vessel damage and consequently poor blood circulation, and other cardiovascular diseases [9]. Further, proper immune cell coordination requires a balance to recruit the appropriate cells but minimize inflammatory tissue damage. Alterations in this balance can render the host incredibly susceptible to infection with opportunistic pathogens [10], and diabetic patients are known to have impaired immune cell recruitment and turnover [11,12,13].

Experimental animal models are frequently used in diabetes research and have the advantage of a controlled experimental system to systematically assess the molecular mechanisms associated with disease [14]. These models fit with many research purposes that are less focused on the autoimmune process and more on hyperglycemia, obesity, or the metabolic syndrome itself. Animal models of diabetes have provided evidence that hyperglycemia is associated with decreased bacterial clearance of GBS, possibly contributing to increased mortality among diabetic animals in sepsis [15,16]. Chemical, approaches such as diabetogenic drugs are often used to induce hyperglycemia in animal models. Several animal species, including the mouse, rat, and monkey, are sensitive to the pancreatic β-cell cytotoxic effects of streptozotocin (STZ). Currently, STZ is most often used to induce diabetes in rats and mice due to its stability, and it is less toxic than other drugs, such as alloxan [17]. STZ-involved diabetic animal models are affordable alternatives to elucidate mechanisms of diabetic pathogenesis and its complications. 

The aim of this study was to examine the impact of diabetes on the severity of invasive GBS infection in the lungs and hearts using an STZ-induced diabetic murine model.

## 2. Materials and Methods

### 2.1. Bacterial Strain and Culture Condition

Two GBS capsular type III strains were used in this study, both belonging to a hypervirulent lineage, ST-17. GBS90356 was isolated from the cerebrospinal fluid of a 3-day-old male neonate with fatal meningitis, and the COH1 strain was isolated from a septicemic newborn. For experiments, both strains were cultured on blood agar base (BAB; Oxoid) plates containing 5% defibrinated sheep red blood cells for 24 h at 37 °C and subcultured in brain heart infusion broth (BHI; Difco) at 37 °C until they reached an optical density (OD_600_) of 0.4 (~1 × 10^8^ colony forming units (CFU) per mL).

### 2.2. Animals and Treatment

Sixty-four male (*n* = 30) and female (*n* = 34) CD-1 mice aged 6–8 weeks weighing 30–35 g were used in this work. Mice were randomly divided into four groups: 1. non-diabetic animals (non-diabetic and non-infected; NDNI), (*n* = 3); 2. non-diabetic infected intraperitoneally with the GBS90356 (*n* = 14) or COH1 (*n* = 15) strain (non-diabetic infected; NDI); 3. streptozotocin (STZ)-induced diabetic (diabetic non-infected; DNI), (*n* = 3); 4. STZ-induced diabetic infected intraperitoneally with the GBS90356 (*n* = 14) or COH1 (*n* = 15) strain (diabetic infected; DI). The mice were given free access to a standard laboratory diet and water. Blood glucose was measured for both male and female mice (i) before the experiments; (ii) after diabetes *mellitus* induction with STZ; and (iii) at the end of the experiments. Animals fasted for 6 h before blood glucose measurements. Animals that were resistant to the STZ treatment were removed from the study. All the procedures were performed under general anesthesia, and all efforts were made to minimize suffering. Some mice infected with the hypervirulent GBS90356 strain were discovered after having already succumbed to infection, and in this instance, tissues were not collected postmortem. Data showing the GBS90356 bacterial burden in tissues are indicative of 8/14 mice where tissues were able to be harvested.

### 2.3. Ethics Statement

This study was carried out in accordance with the recommendations and guidelines adopted by the Brazilian National Council for the Control of Animal Experimentation. The protocol was approved by the Ethical Commission for the care and use of experimental animals, Roberto Alcantara Gomes Biology Institute, Rio de Janeiro State University—UERJ (Permission number: 47/2016).

Animal experiments were also performed at the University of Colorado Anschutz Medical Campus, approved by the Institutional Animal Care and Use Committee (IACUC) protocol #00316 and were performed using accepted veterinary standards. The University of Colorado Anschutz Medical Campus is AAALAC accredited, and its facilities meet and adhere to the standards in the “Guide for the Care and Use of Laboratory Animals”.

### 2.4. Diabetes Mellitus Induction and GBS Systemic Infection

Diabetes *mellitus* was induced by the administration of STZ (Sigma, St. Louis, MO, USA) freshly dissolved in 40 mM citrate buffer (pH 4.5). Mice (DI and DNI animals) were injected intraperitoneally with a single dose of STZ (180 mg per kg bodyweight, 100 µL total volume), while twenty-eight (NDNI and NDI animals) received only citrate buffer with the same volume. After 48 h, blood samples were drawn from the tail of these mice to determine fasting blood glucose level. Glucose levels of more than 200 mg/dL (Accu-Chek Advantage II, Roche Diagnostica, São Paulo, São Paulo, Brazil) were considered diabetic and included in the experiments. Three days after the STZ injection, two groups (DI and NDI animals) were infected intraperitoneally with 1 × 10^7^ CFU/100 µL of GBS90356 or COH1 strain [18]. After five days, mice were euthanized (via CO_2_ asphyxiation and cervical dislocation), and lungs, heart, brain, spleen, and blood were aseptically removed. 

### 2.5. Broncheoalveolar Lavage (BAL) Collection and Measurement of Intracellular ROS

After euthanizing all mice, BAL was performed as previously described by Van Hoecke et al. [19]. Our group adapted the protocol by cannulating the trachea and washing the lungs two times with phosphate-buffered saline (PBS, pH 7.2) using a volume of ~750 µL/wash. The retrieved fluid was kept on ice. The lavage washes were centrifuged at 300× *g* for 5 min at 4 °C. The BAL (~1 mL per mouse) was stored on ice for 2 h prior to intracellular ROS analysis. 

The BAL was incubated in 96-well black-walled plates at a density of 6 × 10^3^ cells/well at 37 °C in a humidified atmosphere of 5% CO_2_ for 2 h. Then, the BAL cells were washed three times with PBS and incubated with the CM-H2DCFDA probe [5-(and-6)-chloromethyl-2,7-dichlorodihydrofluorescein diacetate] for 1 h. Fluorescence intensity was assessed throughout the 180-min period using an Envision microplate reader. ROS production was detected through fluorescence emitted from dichlorofluorescein (DCF) oxidation. Fluorescence was monitored at excitation and emission wavelengths of 495 nm and 525 nm, respectively.

### 2.6. Bacterial Viability Assay

The organs were macerated separately from each animal and diluted 1:1000 in 0.9% saline solution; they were plated in BAB containing 5% sheep red blood cells and incubated at 37 °C overnight. Bacterial colonies were visually counted. Capsular typing and detection of the ST-17 hypervirulent clone were performed by PCR [20]. 

### 2.7. Histopathological Analysis

After necropsy, tissue fragments of lungs and hearts from GBS90356 infected animals were fixed with 4% freshly prepared formaldehyde for 24 h, dehydrated in ethanol series (30% to absolute), diaphonized in xylol and embedded in paraffin. Thin 5 μm sections were stained using four protocols: hematoxylin and eosin (H&E) for general morphological observation; Gomori Trichrome for collagen deposition identification; Giemsa to stain the bacteria (GBS90356); and Weigert Resorcin-Fuschin (WRF) to selectively stain the elastic fibers. The samples were analyzed using the light microscope Olympus (model BX 53) and digital camera with cellSens Entry 1.18 software. Quantification was performed using ImagePro Plus, and statistical analyzes were obtained using GraphPad Prism 5 by one-way ANOVA and Newman-Keuls’s test. ImageJ analysis software was used to quantify immune cell infiltration in twenty stained image sections of each group at 40× magnification.

### 2.8. RNA Purification and qRT-PCR 

A qRT-PCR analysis of several immune marker transcripts was performed using heart homogenates. RNA was purified using the Machery-Nagel Nucleospin kit and treated with the turbo DNAse kit (Invitrogen, San Diego, CA, USA) according to manufacturer instructions. cDNA was synthesized using the SuperScript cDNA synthesis kit (QuantaBio, Beverly, MA, USA) per manufacturer instructions. cDNA was diluted 1:250 to further reduce bacterial DNA contamination, and qRT-PCR was performed using PerfeCTa SYBR Green (QuantaBio, Beverly, MA, USA), BioRad CFX96 Real-Time System, and C1000 Touch Thermocycler. The following primer sequences (shown 5′ to 3′) were used in this study: mouse CD11b (forward [F], ATGGACGCTGATGGCAATACC; reverse [R], TCCCCATTCACGTCTCCCA); mouse CD11c (F, CTGGATAGCCTTTCTTCTGCTG; R, GCACACTGTGTCCGAACTCA); mouse MHCII (F, AGCCCCATCACTGTGGAGT; R, GATGCCGCTCAACATCTTGC); mouse CD3 (F, ATGCGGTGGAACACTTTCTGG; R, GCACGTCAACTCTACACTGGT); mouse F4/80 (F, CTTTGGCTATGGGCTTCCAGTC; R, GCAAGGAGGACAGAGTTTATCGTG); mouse Ly6g (Gr-1) (F, CTTCTCTGATGGATTTTGCGTTG; R, AGTAGTGGGGCAGATGGGAAG); and mouse GADPH (F, CGTCCCGTAGACAAAATGGT; R, TCTCCATGGTGGTGAAGACA). Fold changes in transcript abundance were calculated using ΔΔCT, by which target gene transcript levels were normalized to those of the internal housekeeping gene, GADPH. qRT-PCR data represent the average of three biological replicates.

### 2.9. ELISA

The KC protein and IL-1β in homogenized lungs and heart tissues were quantified using R&D systems ELISA kits (R&D Systems, Minneapolis, MN, USA). The KC protein and IL-1β detected were normalized to tissue weight (pg of protein per mg of tissue).

### 2.10. Statistics

Statistical analysis was performed using Prism version 5.0.0 for Windows 10 (GraphPad Software, La Jolla, CA, USA). The statistical details of the experiments, such as the statistical test used, experimental n, and significance, can be found in each figure legend.

## 3. Results

### 3.1. Glycemia, Body Weight, and Survival Parameters

**Figure 1:**
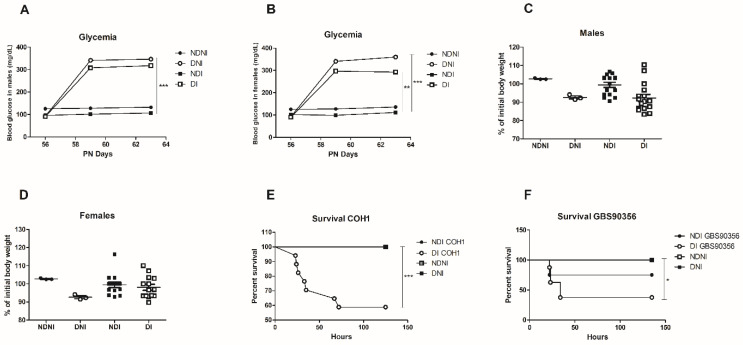
Assessment of glycemia, body weight and survival rates of diabetic and non-diabetic mice after GBS infection. Means of the blood glucose levels in male (**A**) and female (**B**) mice (mg/dL) of normal control (NDNI), non-diabetic infected (NDI), diabetic non-infected (DNI), and diabetic infected (DI) during 5 days of GBS infection; (**C**,**D**) Percentage of initial body weight in both diabetic and non-diabetic groups for male and female mice; (**E**) Kaplan-Meier plot showing survival of diabetic (DI) or non-diabetic (NDI) mice challenged with GBS COH1 strain or GBS90356 (**F**) strain. NDI COH1, NDNI and DNI groups presented 100% survival during the studies. Statistical analysis: (**A**–**D**) One-way ANOVA with Newman-Keuls post-test, (**E**,**F**) Mantel-Cox Log-rank test; * *p* < 0.05, ** *p* < 0.007, *** *p* < 0.001.

Blood glucose levels were significantly higher in STZ-induced diabetic mice when compared to untreated animals (*p* < 0.01). STZ-treated mice presented severe hyperglycemia (>300 mg/dL) in contrast with normoglycemic controls (<109 mg/dL) during similar monitoring times for both male and female animals (Figure 1A,B).

Body weight and food consumption were monitored throughout the experiment. At the end of the experiment, there was no significant weight loss or gain difference between groups for both male and female animals (Figure 1C,D). Despite that, diabetic or non-diabetic animals infected with GBS strains showed a tendency for weight loss.

To assess if diabetic mice were more susceptible to systemic infection with COH1 or GBS90356 strains, we observed if GBS was leading to bacteremia in mice over 5 days. Non-diabetic animals infected with either strain exhibited increased survival compared to diabetic animals (Figure 1E,F). None of the controls (NDNI or DNI) succumbed to infection.

### 3.2. Recovery of GBS COH1 and GBS90356 Strains In Vivo

**Figure 2:**
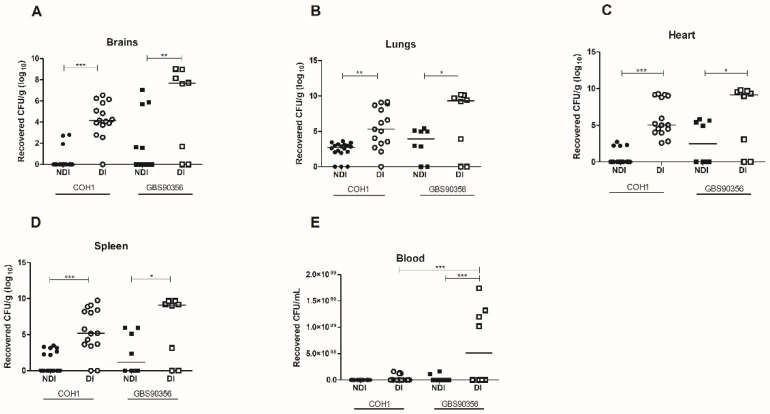
Colony forming units (CFU) in diabetic and non-diabetic mice infected by GBS. (**A**) After 5 days of infection with COH1 (*n* = 15) or GBS90356 (*n* = 8) strains, NDI and DI mice were euthanized, and bacterial loads in brains, (**B**) lungs, (**C**) hearts, (**D**) spleen and (**E**) blood were quantified. Each dot represents the bacterial load of one animal normalized by tissue weight (CFU/g). All tissues presented significantly higher bacterial loads with COH1 and GBS90356 strains in diabetic-infected mice (DI) when compared to non-diabetic-infected mice. Only GBS90356 infected mice presented significant numbers of viable bacteria in the blood after five days of infection. Black and white circles in the figures represent NDI COH1 and DI COH1 infected animals, respectively. Black and white squares represent NDI GBS90356 and DI GBS90356 infected animals, respectively. Statistical analysis: One-way ANOVA with Newman-Keuls post-test. * *p* < 0.05, ** *p* < 0.01, *** *p* < 0.0001.

Viable GBS strains were recovered in pure culture from all organs analyzed, showing bacterial spread to different sites through the bloodstream after intraperitoneal infection (Figure 2). PCR analysis detected GBS on DNA extracted from pure culture for both strains (data not shown). For mice infected with COH1, there was a ~4-fold difference in the number of bacteria recovered from the brains and spleen of NDI and DI animals, while mice infected with GBS90356 had a ~3-fold difference between NDI and DI. A similar phenotype was observed in the heart tissues of mice infected with COH1, with a ~5-fold difference between NDI and DI, and for GBS90356, a ~3-fold difference between NDI and DI. In the lungs, similar ~3-fold increases in CFU were observed in mice infected with COH1 or GBS90356. No viable bacteria were observed in blood samples from non-diabetic infected (NDI) or diabetic infected (DI) mice infected with the COH1 strain. However, the GBS90356 strain showed high bacterial counts in DI animals (~4-fold) (*p* < 0.001) (Figure 2E).

### 3.3. General Aspects of Pulmonary Injury in Diabetic and Non-Diabetic Mice Infected with GBS

**Figure 3:**
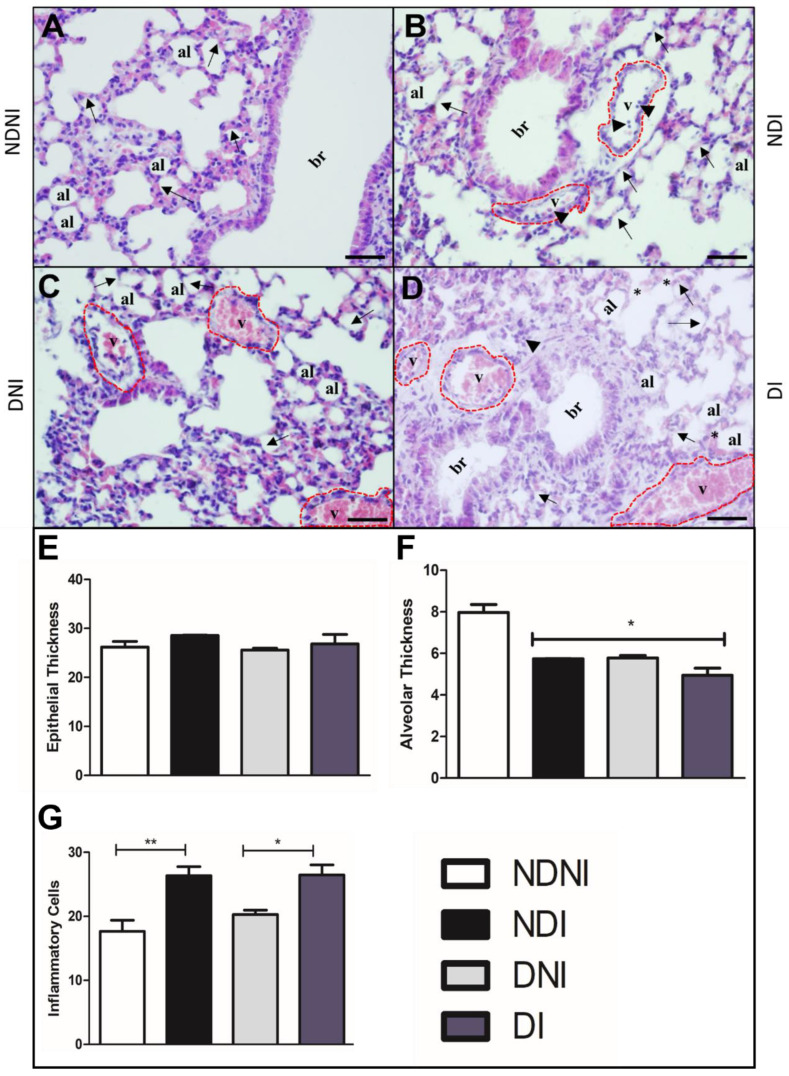
H&E-stained histological section of lungs from diabetic and non-diabetic animals infected or not by GBS strain GBS90356. (**A**) Non-diabetic uninfected group (NDNI) showing normal bronchioles and intact alveolar septa (arrows); (**B**) Non-diabetic group infected with GBS (NDI) present blood vessels congested (red dotted line) with inflammatory cells around the bronchioles (arrowhead) and collapsed septa (arrows); (**C**) Non-infected diabetic group (DNI) showing collapsed septa (arrows), congestive vessels (red dotted line) containing a large number of red blood cells; (**D**) Diabetic and infected by GBS group (DI) showing congested blood vessels (red dotted line), inflammatory cells (arrowhead), regions with collapsed septa (black arrows) and rupture of septa (asterisk); (**E**) Epithelial thickness on the lungs showed no difference between groups; (**F**) Alveolar thickness was significantly lower in DNI, NDI and DI animals; (**G**) Immune cell infiltration showing a higher number of general inflammatory cells in both infected groups (*NDI compared to NDNI; **DI compared to DNI). v—blood vessel lumen; br—bronchioles; al—pulmonary alveolus. Scale bar: 50 µm. ImagePro Plus and GraphPad Prism were used for analysis and quantification. Statistical analysis: (**E**–**G**) One-way ANOVA with Newman-Keuls post-test; * *p* < 0.05, ** *p* < 0.007.

To further investigate general aspects of the lungs and possible tissue damage induced by GBS infection, we stained lung sections of naïve or GBS90356 infected diabetic or non-diabetic animals using H&E, Weigert, and Gomori Trichrome. Histological sections of the lungs of non-diabetic and non-infected animals (NDNI) stained with H&E showed normal microvilli and intact alveolar septa (Figure 3A), while non-diabetic infected (NDI) presented congested blood vessels with inflammatory cells around the bronchiole and collapsed septa (Figure 3B). Sections from diabetic non-infected (DNI) animals showed regions with collapsed septa and evidence of congested vessels (Figure 3C). The diabetic and infected group (DI) showed congested vessels, inflammatory cell infiltrate, collapsed septa, and red blood cell extravasation (Figure 3D). Blood vessel congestion was observed in diabetic groups, infected or non-infected (Figure 3C,D). No groups presented significant differences in pulmonary epithelial thickness (Figure 3E); however, the alveolar wall thickness was significantly reduced in NDI, DNI and DI groups as compared to the NDNI group (Figure 3F; *p* < 0.05). The number of pulmonary inflammatory cells was higher in infected groups (NDI and DI) when compared to non-infected animals (Figure 3G; * *p* < 0.05, ** *p* < 0.007).

**Figure 4:**
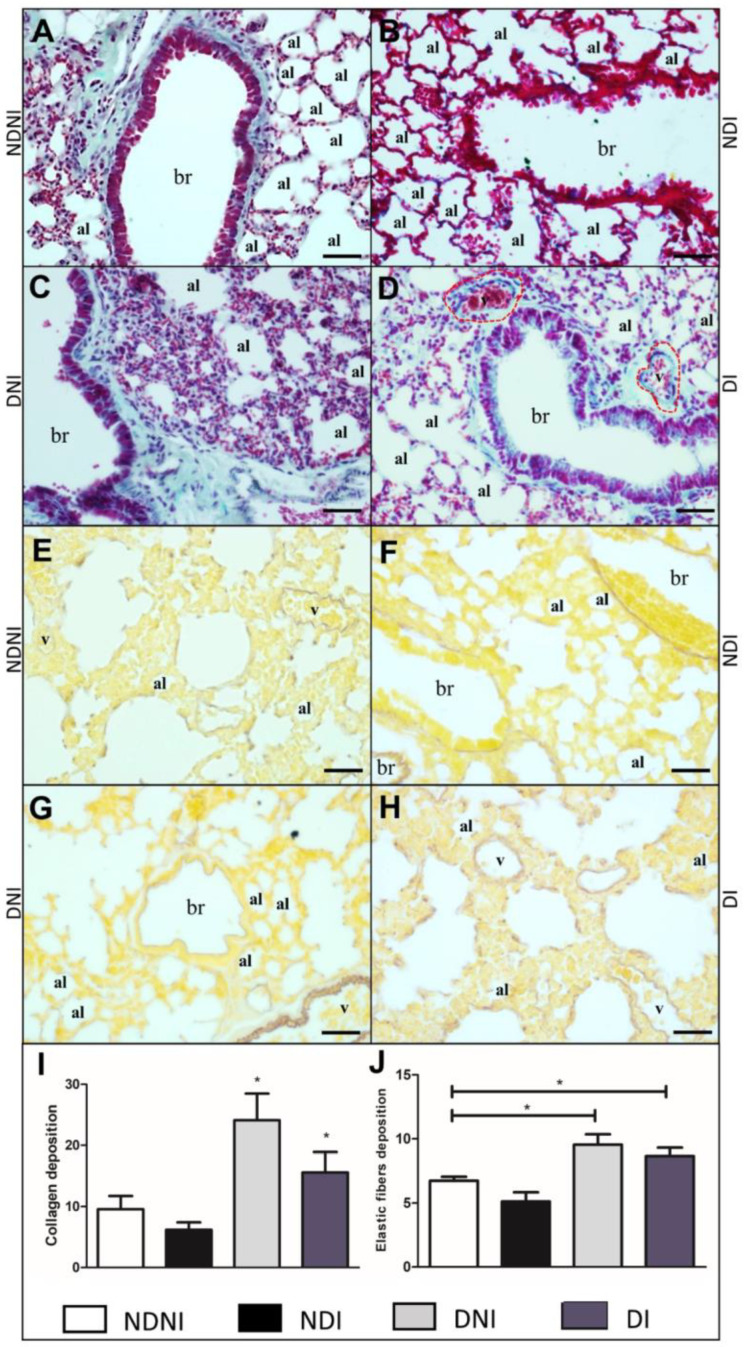
Histological section of lungs from diabetic and non-diabetic animals that were uninfected or infected by GBS strain GBS90356. Gomori Trichrome staining of collagen fibers in blue/green (**A**–**D**) and Weigert staining elastic fibers deposition in brown (**E**–**H**). (**A**) Non-diabetic and non-infected group (NDNI) showed collagen deposition in the parenchyma and around the bronchioles; (**B**) Non-diabetic infected by the GBS group (NDI) presented a reduction of collagen fibers in the parenchyma; (**C**) Diabetic and non-infected animals (DNI) exhibited increased collagen fibers deposition in the parenchyma and around the bronchioles; (**D**) Diabetic and infected group (DI) displayed collagen deposition around the bronchioles and congested blood vessels; (**E**) NDNI showed a thin layer of elastic fibers deposition around the blood vessels; (**F**) Elastic fiber deposition around the bronchioles in NDI group; (**G**) DNI animals showed elastic fibers deposition around the bronchioles and blood vessels; (**H**) Increased elastic fibers around blood vessels and the lung parenchyma in DI group. Both DNI and DI animals presented an increase in collagen deposition ((**I**); *p* < 0.004) and in elastic fibers when compared to NDNI and NDI groups ((**J**); *p* < 0.001). Collagen fibers were stained in blue/green. Elastic fibers were stained brown. ImagePro Plus was used for quantification based on pixel colors of 15 images of each animal in all groups in this study after staining. Red dotted line—periphery of blood vessel; v—blood vessel lumen; br—bronchioles; al—pulmonary alveolus. Scale bar: 50 µm. Statistical analysis: (**I**,**J**) One-way ANOVA with Newman-Keuls post-test; * *p* < 0.05.

Non-diabetic and non-infected animals presented normal lung histology (Figure 4A), and the lung of the DNI (Figure 4C) or DI (Figure 4D) exhibited an increase in collagen fiber deposits, mainly in the lamina propria (Figure 4C) when compared to the NDNI animals (Figure 4A,B). Collagen deposition and elastic fiber alterations were not identified in NDNI and NDI groups (Figure 4B,E,F). A significant increase in elastic fibers was observed around the bronchioles and between the interalveolar septa in DNI (Figure 4G) and DI (Figure 4H) groups, suggesting an increase in collagen deposition (Figure 4I; *p* < 0.01) and elastic fibers (Figure 4J; *p* < 0.001) associated with a diabetic condition, independent of bacterial infection.

### 3.4. Cardiac Lesions in Diabetic and NON-Diabetic Mice Infected with GBS

**Figure 5:**
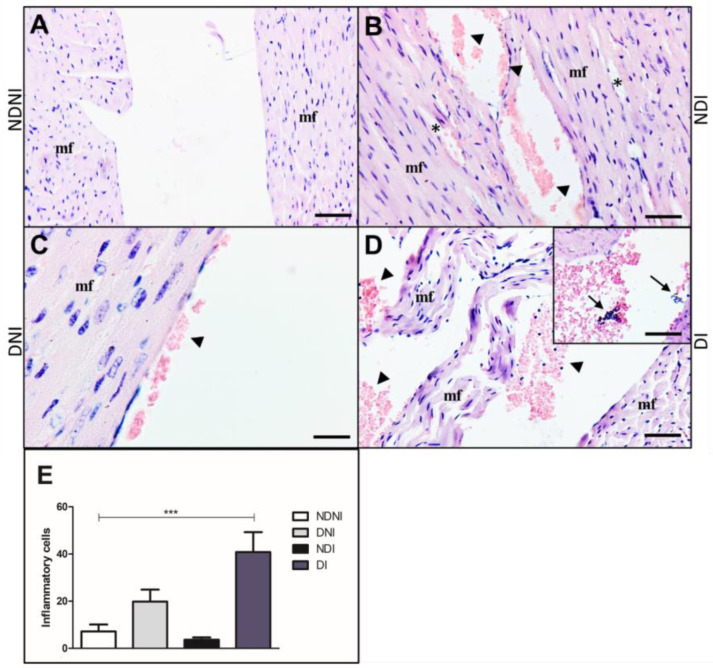
Histological section of left side of the heart from diabetic and non-diabetic animals that were uninfected or infected by GBS strain GBS90356 stained with Giemsa. (**A**) NDNI group showed a healthy heart with intact muscle fibers (mf); (**B**) NDI animals presented rupture (asterisk) of the muscle fiber (mf) and red blood cells adhered on the left-sided valve wall (arrowhead); (**C**) DNI exhibited the left valve with intact muscle fibers and a small number of red blood cells adhered to the valve wall (arrowhead); (**D**) Diabetic infected with GBS group (DI) showed extensive damage with disorganization of the muscle fibers (mf) of the left-sided valve, a large number of red blood cells and inflammatory cells adhered on the valve wall (arrowhead). Inset: Bacterial aggregates in left ventricle tissue (arrows); (**E**) immune cells infiltration showing a higher number of general inflammatory cells in diabetic infected groups when compared to NDNI, NDI and DNI groups (***). Scale bars: 50 µm ((**A**,**B**,**D**,**E**) and Inset) and 20 µm (**C**). ImagePro Plus was used for cell quantification. Number of inflammatory cells obtained by quantification of 20 sections of each group, 40× magnification. Statistical analysis: (**E**) One-way ANOVA with Newman-Keuls post-test; *** *p* < 0.001.

Histological analyses of Giemsa stained-cardiac left-sided valves in NDNI showed muscle fibers organization without damage, cellular infiltration, or cells attached to the heart wall (Figure 5A). In NDI animals, alterations in muscle disorder fibers in the left-sided valve were observed (Figure 5B). Interestingly, in the DNI group, we observed red blood cells adhered to the valve wall (Figure 5C), and in the DI group, we observed tissue disorganization with red blood cells adhered to the valve and inflammatory cell infiltrate and bacterial aggregates in the left ventricle tissue (Figure 5D and Inset). DI mice also presented a higher number of inflammatory cells in the valves when compared to other groups (NDNI, NDI and DNI, respectively; Figure 3E, *p* < 0.001). 

### 3.5. Proinflammatory Cytokine Production, Immune Cell Infiltration, and ROS Production in Diabetic and Non-Diabetic Mice Infected with GBS

**Figure 6:**
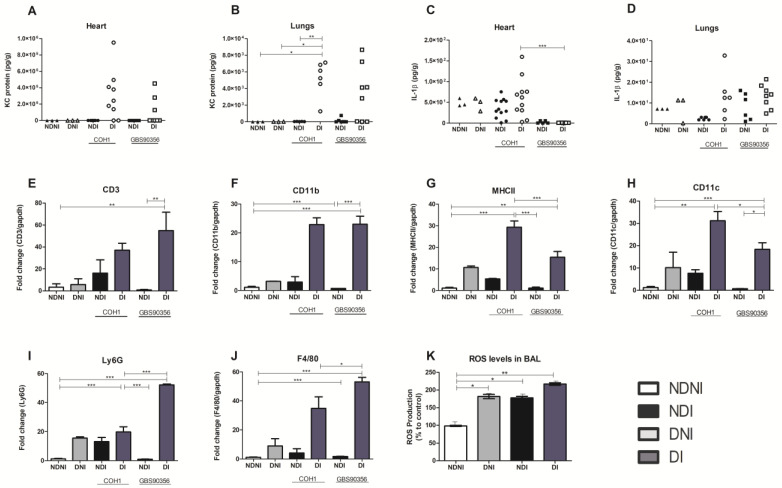
Pro-inflammatory cytokine release, immune marker expression, and ROS expression in diabetic and non-diabetic mice infected or not with GBS. ELISA was performed on mouse heart and lung tissue homogenates to assess cytokine protein levels. KC (**A**,**B**) and IL-1β (**C**,**D**) were quantified for both organs from diabetic or non-diabetic mice challenged with either COH1 or GBS90356 strains. (**E**–**J**) Quantification of immune marker transcripts in diabetic or non-diabetic mice infected or not with COH1 or GBS90356 strains. The presence of CD3, CD11b, MHCII, CD11c, Ly6G and F480 transcript was determined using qRT-PCR. (**K**) ROS expression was determined after BAL extraction from NDNI, DNI, NDI (infected with GBS90356) and DI (infected with GBS90356) and treatment with CM-H2DCFDA probe for 1 h. DI infected mice produced higher levels of ROS when compared to NDNI, DNI and NDI groups. Black and white circles in the figures (**A**–**D**) represent NDI COH1 and DI COH1 infected animals, respectively; black and white squares represent NDI GBS90356 and DI GBS90356 infected animals, respectively. NDNI and DNI animals are represented with black and white triangles, respectively. Statistical analysis: One-way ANOVA with Newman-Keuls post-test. * *p* < 0.05, ** *p* < 0.01, *** *p* < 0.0001.

GBS has a highly invasive potential and can especially cause an excessive inflammatory environment, sepsis, and death at the beginning of life, in the elderly, and in diabetic patients [21]. An increased and uncontrolled immune cell recruitment after bacterial infections is critical during diabetes due to the ability of these cells to produce cytokines and high levels of reactive oxygen species [22]. Acknowledging this, we wanted to quantify protein levels related to neutrophil and macrophage recruitment in heart and lung tissue. We observed that DI animals had higher amounts of neutrophil chemokine KC in both tissues when compared to other groups (Figure 6A,B) as determined by ELISA. However, it was only statistically significant in the lungs of diabetic animals infected with the COH1 strain. The cytokine IL-1β was also present in both the lungs and heart, and although we did not observe differences between groups in the lungs (Figure 6D), COH1 infection induced more IL-1β in the cardiac tissue of diabetic animals when compared to other treatment groups (Figure 6C). KC protein and IL-1β levels were normalized to the bacterial CFU within the same tissue.

To assess immune cell signaling and characterize cellular presence in cardiac tissues, we selected a panel of genes to cover a range of immune cells (CD11b, Ly6G for neutrophils; CD11b, F4/80 for macrophages; CD11c, MHCII for dendritic cells; CD3 for T cells). The high presence of the CD3 gene was verified in diabetic mice infected with GBS90356 strains when compared to all other groups (Figure 6E). CD11b and F4/80 had significantly higher expression in both diabetic infected groups when compared to all others, suggesting that both strains may contribute to a macrophage response at 5 days of infection (Figure 6F,J).

In both diabetic-infected groups, MHCII and CD11c also showed significantly higher expression compared to other groups, with COH1 diabetic-infected animals showing a higher expression of markers for dendritic cells when compared to GBS90356 diabetic-infected animals (Figure 6G,H). Ly6G in GBS90356 diabetic-infected animals was higher than all other groups, including COH1 diabetic-infected mice.

Because we saw an indication of increased immune cell presence in diabetic-infected mice, we hypothesized that GBS would also encounter increased ROS production. Levels of ROS generation were evaluated during GBS infection in diabetic or non-diabetic animals, as oxidative stress has been linked to a myriad of pathologies and is a marker of inflammation. We evaluated ROS production with a probe treatment on cells obtained from murine BAL. Compared to NDNI, ROS production was higher in NDI, DNI and DI animals. In general, levels of ROS were highest in diabetic-infected animals when compared to other groups (Figure 6K, *p* < 0.01). GBS strains could elicit more inflammation in diabetic mice tissues compared to the non-diabetic (infected or control) mice. This may contribute to lower survival observed between groups during systemic infection (Figure 1E,F).

## 4. Discussion

GBS is a recognized pathogen associated with infections in newborns, pregnant and puerperal people. However, an increasing incidence of invasive diseases by this pathogen has been reported in patients with immunocompromising diseases, such as diabetes *mellitus* [23]. Human diabetic patients reportedly have more susceptibility to GBS bacteremia due to defects in phagocytosis and microbial killing [24,25]. The hyperglycemic environment favors immune dysfunction, angiopathies, neuropathy, a decrease in the antibacterial activity of urine, and gastrointestinal and urinary dysmotility, which collectively contribute to morbimortality in diabetic patients [26]. 

To date, few studies have assessed the influence of diabetes in enhancing susceptibility to GBS bacteremia. Edwards and Fusilier [16] described the higher vulnerability of diabetic mice infected by GBS, associated with bacteremia and clearance mediated by the reticuloendothelial clearance system. In 2015, others showed that the organs of diabetic adult mice were less colonized with GBS after immunization with glyceraldehyde-3-phosphate dehydrogenase (GAPDH), a glycolytic enzyme detected at the bacterial surface [17]. In addition, the incidence of invasive GBS infections among patients with poorly controlled diabetes was four-fold higher than among patients with well-controlled diabetes [27].

In the present work, streptozotocin (STZ)-diabetic animals exhibited elevated blood glucose (>300 mg/dL) in a sex-independent manner. Data also showed that in five days, there was no significant difference in weight change, but DI animals with both GBS strains had a lower rate of survival (60% for COH1 DI mice compared to 100% survival of NDI and 40% for GBS90356 DI mice compared to 70% survival of NDI). Future studies will aim to further investigate later time points of survival. The animals included in this study were used to assess the pathological consequences of diabetes during intraperitoneal infection by GBS. Intraperitoneal routes have been commonly used as an efficient model of systemic infection of several bacterial species [28,29,30]. The intranasal route, despite biologically relevant in the context of neonatal colonization with GBS following passage through the birth canal, is not very well explored as a route in adult animals. In this study, we aimed to understand how GBS impacts diseases and pathological injuries in other organs in the context of diabetes.

The intraperitoneal route of infection is already established for GBS, generating a systemic infection that will reach multiple organs, as shown in Figure 2. According to previous publications, this route has been used as a standard practice in several systems, such as the urinary tract or the intestinal mucosal [18,31], and we have shown here that CD-1 mice succumbed rapidly to GBS infection through this route. After intraperitoneal inoculation, the microorganism progressively disseminated through the bloodstream into various target organs, such as the heart, lungs, brain, and spleen. The pure culture was detected in all organs, and GBS was identified by PCR. Statistical analysis showed a difference in the viable bacteria count for all organs of diabetic and non-diabetic animals, and especially for the lungs, there was evidence of structural differences and differences in inflammatory signals. Signs of lung injury were previously demonstrated in a primate model of GBS infection, including histologic changes such as accumulation of inflammatory cells, evidence of necrosis, inflammatory-related tissue thickening, collapsed septa, fibrin exudation, or hemorrhage [32]. Presently, pronounced histologic evidence of lung inflammation and injury was verified in DI animals. Diabetic animals presented intra-alveolar hemorrhage and collapsed septa. Moreover, the diabetic mice infected by GBS also showed flooding of the alveolar space with fluid and intense red blood cell extravasation. Similar pathological alterations were observed in a diabetic patient with septicemia caused by GBS, where the authors described a focal area of parenchymal honeycombing and hemorrhage characterized by septal thickening and bronchiolization of alveolar spaces [33].

The extracellular matrix (ECM) is a dynamic three-dimensional fibrous network essential to the mechanical properties of the airways [34]. They are responsible for modulating bronchoconstriction, airway reopening and the remodeling process [35,36]. In the present study, collagen and elastic fiber content increased in the airways of diabetic animals with or without GBS infection. This is consistent with the experimental findings where infectious pulmonary granulomas showed a significantly higher number of collagen and elastic fibers [37], suggesting a protective effect on the airways. Thus, ECM proteins seem to be fundamental to an effective response to infection. However, currently little is known of the functional consequences of altered collagen and elastin content within the airway wall after GBS infection, mainly in diabetic patients.

The production of proinflammatory cytokines may be crucial in the rupture of cell membranes by GBS, such as IL-1β and TNF-α [38]. Their roles may include the upregulation of matrix metalloproteinase production, principally MMP-9, which is known to participate in diabetic wound healing. In the pathogenesis of diabetic wounds, MMP-9 impairs the balance of ECM synthesis and degradation, leading to unhealed wounds. The authors also showed that collagen fibers were less dense and disordered after incubation with plasma exposed to heat-inactivated GBS, suggesting the existence of a virulence factor that maintains its activity independently of bacterial viability [39]. Thus, the metalloproteases induced by GBS in diabetic animals may be related to a reduction in collagen fibers when compared with diabetic non-infected groups. 

In this investigation, we also showed a higher number of viable bacteria in the spleen and heart of diabetic animals, suggesting an increased risk of invasive disease. Interestingly, the tilapia spleen is one of the main target tissues during GBS infection [40]. The spleen represents an important immune organ, as it plays a vital role in coping with diseases. If the tilapia spleen, which is involved in the hematopoiesis process, is affected with microlesions after infection, this may cause irreversible problems such as the sudden death of the animal [39]. Additionally, the first case of GBS endocarditis in a healthy adult patient complicated by multiple mycotic aneurysms of the hepatic artery was previously described [41]. GBS infective endocarditis is an unusual cause and frequently affects patients with debilitating diseases. A previous study showed that endocarditis in the left-sided valves due to GBS was more serious than that caused by other species of *Streptococcus* spp., with multiple complications and increased mortality [42]. The most frequent comorbidity was diabetes *mellitus* in GBS left-sided infective endocarditis. Currently, our data show that diabetic mice were more susceptible to GBS infection than non-diabetic mice, showing bacterial aggregates in cardiac tissue. Histological sections imaged by Batista e Ferreira [34] described acute inflammatory infiltrate, hemorrhage, and several bacterial colonies in an area of acute myocardial infarction in a diabetic patient who succumbed to GBS infection.

The KC protein (CXCL-1 in humans) plays an important role in the development of inflammation as a chemotactic factor for neutrophils [42]. In our study, KC protein levels were higher in DI groups compared to non-diabetic uninfected, non-diabetic infected with both GBS strains and diabetic uninfected groups, suggesting a more robust infection and increased inflammation in diabetic-infected mice. This was also corroborated by IL-1β in the heart, although it was only significantly higher in COH1-infected diabetic animals. Interestingly, this cytokine was not expressed in different levels between groups in lung tissue, suggesting a potential tissue-specific response during diabetic GBS infection. IL-1β has been shown to be protective in several bacterial infection models by promoting rapid recruitment of neutrophils to inflammatory sites, induction of cytokines and chemokines and stimulation of Th17 responses [43]. However, several inflammation-associated human pathologies can also be primarily driven by unrestrained IL-1β production [43].

It is important to characterize immune cell presence in different tissues, to understand how the host responds to an invading pathogen, and to determine if this response is successful in eliminating the microorganism. In our study, according to the markers identified by qRT-PCR, macrophages, neutrophils, and dendritic cells may be presented at higher numbers in diabetic-infected animals when compared to other groups. Expression of T cell marker CD3 was significantly higher in diabetic animals infected with the GBS90356 strain compared to the animals infected with the COH1 strain. Acute, short-term hyperglycemia affects all major components of innate immunity and impairs the ability of the host to combat infection [44], and our study shows a potential impact of diabetic infection on T cell responses as well. Further investigation is required to understand if immune responses to GBS infection are impaired by hyperglycemia and how GBS may evade these responses.

The continuous identification of invasive diseases among diabetic patients highlights the need for appropriate prevention strategies. Our data showed the importance of GBS as a cause of severe infection in patients with diabetes, as well as the high risk of fatal outcomes. We demonstrated that hyperglycemic animals were more susceptible to GBS infections and that two different strains could disseminate and persist in several tissues. GBS strains also showed to induce a hyperinflammatory response that is enhanced in diabetic murine lungs and hearts and leads to histopathological lesions in both tissues. These data also support the need to determine specific contributions of ECM components to airway function and their possible significance in the general progression of the disease in diabetic patients infected by GBS.

## Data Availability

Not applicable.
